# Potential bio-indicators for assessment of mineral status in elephants

**DOI:** 10.1038/s41598-020-64780-0

**Published:** 2020-05-15

**Authors:** Fiona Sach, Ellen S. Dierenfeld, Simon C. Langley-Evans, Elliott Hamilton, R. Murray Lark, Lisa Yon, Michael J. Watts

**Affiliations:** 1 Inorganic Geochemistry, Centre for Environmental Geochemistry, British Geological Survey, Nicker Hill, Keyworth, Nottingham, United Kingdom; 2 School of Biosciences, University of Nottingham, Sutton Bonington, United Kingdom; 3 Ellen S. Dierenfeld, LLC, Saint Louis, MO 63128 USA; 4 School of Animal, Rural & Environmental Sciences, Nottingham Trent University, Southwell, United Kingdom; 5 School of Veterinary Medicine and Science, University of Nottingham, Sutton Bonington, United Kingdom

**Keywords:** Biomarkers, Zoology, Animal physiology

## Abstract

The aim of this study was two-fold: (1) identify suitable bio-indicators to assess elemental status in elephants using captive elephant samples, and (2) understand how geochemistry influences mineral intake. Tail hair, toenail, faeces, plasma and urine were collected quarterly from 21 elephants at five UK zoos. All elephant food, soil from enclosure(s), and drinking water were also sampled. Elemental analysis was conducted on all samples, using inductively coupled plasma mass spectrometry, focusing on biologically functional minerals (Ca, Cu, Fe, K, Mg, Mn, Na, P, Se and Zn) and trace metals (As, Cd, Pb, U and V). Linear mixed modelling was used to identify how keeper-fed diet, water and soil were reflected in sample bio-indicators. No sample matrix reflected the status of all assessed elements. Toenail was the best bio-indicator of intake for the most elements reviewed in this study, with keeper-fed diet being the strongest predictor. Calcium status was reflected in faeces, (p 0.019, R^2^ between elephant within zoo - 0.608). In this study urine was of no value in determining mineral status here and plasma was of limited value. Results aimed to define the most suitable bio-indicators to assess captive animal health and encourage onward application to wildlife management.

## Introduction

Formulation of an appropriate zoo diet requires husbandry skills and applied nutritional science^1^. Although there is limited agreement in the literature, the use of appropriate bio-indicators to assess elemental status was suggested by Combs *et al*.^2^ to support evidence-based zoo diet assessment. Zoos in the United Kingdom have a responsibility to provide appropriate nutrition to all animals within their care^3^ to prevent nutritional-related disease, compromised welfare and potential reproductive failure. Limited information exists for estimated mineral requirements of elephants^4^, with cases of specific mineral deficiency documented. Due to elephants’ low growth rate and large size, clinical signs of nutrient deficiency may go unnoticed for long periods of time^5^, making nutritional evaluation challenging.

Jansman and Pas (2015)^6^ defined mineral status as the balance between dietary intake of a nutrient and its requirement in the body. Twenty-eight “essential” mineral elements have known metabolic roles in the mammalian system, for which dietary deficiency will lead to clinical deficiency. These include calcium (Ca), phosphorus (P), magnesium (Mg), selenium (Se) and zinc (Zn)^7,8^. Minerals are utilized within the body in various forms or individual compartments, with a central reserve or interchange compartment, usually blood and one or more storage compartments, usually bone or liver. Element and animal species affects the speed of mobilisation of the mineral between compartment(s)^7,8^. Mineral status can also be altered by interactions between dietary components; for example an increase in dietary P causes a decrease in serum Ca^9,10^, and variations in individuals’ metabolism, circadian patterns and pathological state.

### Analysing elemental status in elephants

No single sample matrix exists for analysing elemental status in elephants or for other mammalian species. Circulating blood fraction concentrations, and/or liver tissues have provided the standards for assessment criteria, with limited evidence as to the accuracy of reflection of elemental status in an animal^11^. Having a non-invasive method to assess elephant elemental status would enable diet evaluation, regular assessment of mineral status within the animal and the development of more accurate reference ranges for the species. The practically available sample matrices for this include plasma, toenail and tail hair, urine and faeces.

Blood samples have been significantly correlated to nutritional status in livestock for trace minerals such as copper (Cu), cobalt (Co) and Zn, as determined by health status^6,12^. However, mineral concentrations in the body are under homeostatic control, even when intake is insufficient^13^. Fluctuations in dietary intake may affect plasma mineral levels too slowly or too rapidly to demonstrate true nutritional status in the animal^12,14,15^. Blood sample collection requires elephant and keeper training^16^ and sample storage outside the laboratory may be problematic. Urine samples indicate excesses of certain minerals that have been absorbed, potentially metabolised, and then excreted. They can be useful in determining Ca, iodine (I) and arsenic (As) status, however, a single sample from an individual is insufficient to reflect elemental status, due to homeostatic controls within the mammalian system and variability in hydration status affecting solute concentrations^14,17–19^. Collection from elephants can be problematic as samples must be collected mid-flow without substrate contamination.

Toenail and tail hair samples reflect longer term patterns of dietary intake, over weeks or months^20,21^. Human toenails have been shown to reflect dietary Se levels, and to correlate with blood plasma levels^22^. Additionally, both sample matrices have been used to identify exposure to toxic trace elements such as As^23–25^. Sample collection is less invasive than for blood samples and storage in the field, handling and health and safety is easier. Faecal samples reflect unabsorbed dietary minerals, as well as those re-excreted into the intestines as excess^26^. Separating these relative component contributions in faecal mineral contents is challenging. Elephants consume diets of mixed digestibility, thus less digestible components, including minerals, could be over-represented in samples^21^. However, faecal samples are inexpensive and non-invasive to collect, in both captive and wild elephants.

From existing evidence, there is no recognised sample matrix for assessing mineral status in elephants, other than by using samples of various sample matrices that will differ in suitability for different elements. The approach proposed in this study attempted to model the different sample matrices in terms of measured intake, focussing on minerals essential for health, including Ca, Cu, iron (Fe), potassium (K), Mg, manganese (Mn), sodium (Na), P, Se and Zn and trace metals including As, cadmium (Cd), lead (Pb), uranium (U) and vanadium (V). The overarching aim of this study was to identify the most suitable bio-indictor to reflect elemental intake, and thus elemental status in elephants.

## Materials and Methods

### Statement of ethical approval

(i) Ethical approval was obtained from the University of Nottingham School of Veterinary Medicine and Science Clinical Ethical Review Panel (Reference: 1499,150622) prior to commencing the study.
(ii) All experiments were performed in accordance with relevant UK guidelines and regulations and appropriate permission obtained from each participating zoo.

### Site and elephant selection

The study was conducted between March 2016 and July 2017, using 21 elephants at five UK zoos, as detailed in Table 1. Each zoo was visited four times over one year throughout the study period to account for potential seasonal variation within the keeper-fed diet and in pasture grazing. Zoos were selected to provide a geographical spread across the UK so as to have regional geochemical variation in soils. Approximately equal numbers of each elephant species were included in the study, with only adult elephants selected (over 10 years old). Animals included in the study represented approximately 40% of the total UK zoo elephant population, sampled from 55% of zoos holding more than one elephant^27^.

**Table 1:** Species and sex (male/female) split for UK study elephants, dates for UK zoo visits.

	Species	No. animals (male/female)	Visit 1	Visit 2	Visit 3	Visit 4
Zoo A	*L. africana*	0/4	March 2016	June 2016	Sept 2016	Jan 2017
Zoo B	*E. maximus*	0/4	June 2016	Sept 2016	Jan 2017	April 2017
Zoo C	*E. maximus*	1/6	Sept 2016	Feb 2017	May 2017	August 2017
Zoo D	*L. africana*	1/3	Sept 2016	Jan 2017	April 2017	July 2017
Zoo E	*L. africana*	2/0	Sept 2016	Jan 2017	May 2017	July 2017

### Sample collection

Samples of all offered food items, soil and water available to the elephants, elephant tail hair, toenail, plasma, faeces and urine were collected from each study zoo as summarised in Supplementary Information, Table 1.

### Sample preparation

Soil samples were air-dried, crushed and sieved to ≤2 mm and further milled to ≤40 µm in an agate ball mill (Retsch, Germany). Water samples were filtered with a hydrophilic 25 mm Minisart filter in the field and acidified with 1% HNO_3_ and 0.5% HCl. Elephant food samples and faecal samples were freeze dried, and passed through a food blender as described by Watts *et al*.^28^. Elephant tail hair and toenail samples were cleaned as described in Middleton *et al*.^25^. Although this method was developed for human hair and toenails, due to the similarities in the sample composition, it was applicable to use with elephant hair and toenails. Blood samples were collected as described in Bourne (2005)^16^, using Vacutainer heparin 10 ml collection tubes. Blood samples were mixed by inversion of the sample 10 times immediately after collection; plasma was immediately separated by centrifugation at 1500 g for 10 minutes and transferred into trace element-free tubes (up to 5 × 1 ml aliquots for each sample) at each individual zoo. Samples were transported on ice and frozen at −20 °C upon return to the laboratory.

### Sample digestion for ICP-MS analysis

Soil samples and elephant faecal samples (0.25 g) were digested in a mixed acid solution (HF: 2.5 ml/HNO_3_:2 ml/HClO_4_:1 ml/H_2_O_2_:2.5 ml) on a programmable hot block; 0.5 g of elephant food samples were digested in HNO_3_:10 ml/H_2_O_2_:1 ml mixed solution in a closed vessel microwave heating system (CEM MARS Xpress, USA) as described in Watts *et al*.^28^. In summary, food samples were heated to 100 °C over 5 minutes with 10 ml HNO_3_, held for 1 minute and then heated to 200 °C over 5 minutes and held for 15 minutes. The vessels were then cooled and 1 ml of H_2_O_2_ added to the solution, allowed to settle for 30 mins before repeating the heating cycle, but with the latter stage held at 200 °C for 25 minutes. Sample digests were then diluted to an appropriate acid matric content for ICP-MS analysis. Elephant tail hair samples and elephant toenail samples (0.1 g sample) were digested also using the microwave heating system, but with HNO_3_:4 ml/H_2_O_2_:1 ml and with only one heating cycle, heated to 100 °C over 5 minutes, held for 1 minute and then heated to 200 °C over 5 minutes and held for 30 minutes as described in Middleton *et al*.^25^. Urine samples were diluted 1-in-10 with 2% HNO_3_ prior to analysis as described in Middleton *et al*.^17^. Plasma samples were diluted 1-in-20 with 0.5% HNO_3_ prior to analysis as described in, Phiri *et al*.^29^.

### Elemental analysis

Elemental analysis was conducted on all prepared samples by inductively coupled plasma mass spectrometry (ICP-QQQ; Agilent 8900×, USA) using collision reaction cell mode (reactive gas modes: H_2_ for Se at 7.0 ml/min, O_2_ for As at 30%, He for all remaining elements at 5.1 ml/min) for a suite of 55 elements using internal standards (Sc, Ge, Rh, In and Ir) for drift correction. ICP-MS operating parameters were: Rf power 1550 W; plasma gas flow 15 L/min; carrier gas flow rate 1.0 ml/min. Fifteen biologically functional elements that are routinely used for health assessment were selected for this study, Ca, Cu, Fe, K, Mg, Mn, Na, P, Se, Zn, As, Cd, Pb, U and V. Internal and external analytical quality controls were used including appropriate certified reference materials, selected based upon the sample matrix. Sample blanks were run to determine practical Limit of Detection (LOD, 3*STDEV).

Additionally, soil pH was measured using 10 g soil and 25 ml CaCl_2_ and organic matter content was estimated for soil and faecal samples using loss on ignition (LOI) at 450 °C for 1 g of sample, as described in Watts *et al*.^28^. For normalisation across urinary samples, urinary creatinine was determined using the JAFFE method^30^.

### Analytical quality control

The accuracy of the elemental analysis was verified by analysing the following Certified Reference Materials (CRM):

- Human Hair (GBW09101, China)
- Spinach leaves (SRM1570A, NIST, USA)
- Tomato leaves (SRM1573A, NIST, USA)
- Seronorm Trace Elements Urine L-1 (Sero AS, Norway)
- Seronorm Trace Elements Plasma L-1 (Sero AS, Norway)
- Basalt rock (BCR-2 United States Geological Survey, USA)
- Soil (SRM2711a, NIST USA)
- Soil (BGS 102, British Geological Survey, UK)
- In house toenail (BAPS 2014)

The concentrations of all reference materials were found to be accurate within an acceptable percentage of the certified values for all elements studied here (average % recovery = 97% ± 20, see Supplementary Information Table 2 for keeper-fed diet CRM data and Supplementary Information Table 3 for all other CRM data).

### Input sampling and analysis

Quantities of all food items which were presented to each elephant in their keeper-fed-diets on the day of sample collection were recorded, and water consumption was estimated at 200 litres per elephant per day based on the literature^11,31,32^ details of keeper-fed diets are in Supplementary Information Tables 4–8. These data were entered into Zootrition software^33^ along with elemental analysis data (nutritional breakdown of feed items in Supplementary Information Table 2 keeper-fed diet analysis) to give an estimation of elemental intake in keeper-fed diet for each individual at each sampling point in time.

Elemental intake was estimated from diet, grass and water consumption for each individual elephant to give a combined input value for each element. Grass consumption from pasture grazing for each season was approximately estimated from total dry matter (DM) intake per elephant, based on individual body weight for that season^34^, as shown in Supplementary Information Table 9. Soil consumption could not be estimated and thus was not included in the combined input value. Therefore, intake values may be considered a conservative underestimate.

### Statistical analysis

The objective of the analysis was to assess the evidence that particular measures of intake (e.g. soil, water, keeper-fed diet) for each element were predictive of measured status in a particular sample matrix (e.g. toenail, tail hair, blood, faeces, urine). This was done using a linear mixed model^8^ using R software, version 3.5.0^35^; the outcome of interest was the elemental level in elephant sample matrices (as predicted by inputs). In all models, species and sex were included as fixed effects. Zoo, individual and season were treated as nested random effects, accounting for correlations between individuals in the same zoo, and between repeated observations on the same individual. The linear mixed model (lmm) was fitted with the lme procedure from the nlme library for the R platform^36,37^.

The evidence that combined input was predictive of element status was assessed by fitting a lmm, with species, sex and combined input as the fixed effects. The models were fitted by residual maximum likelihood. The anova.lme command was applied to test the null hypotheses that the fixed effects on the model were zero. The sequential (default) option was used so that the test on the last-named fixed effect (combined input here) is a test of the null hypothesis given that the other fixed effects were already included in the model.

Next, the separate measures of intake were considered as potential predictors of status by a sequential modelling process. First, and without any reference to data, the measures were ordered from the one regarded as most likely to be predictive to the one least likely. The order of predictors was keeper-fed diet, grass, water and lastly, soil.

The first predictor in this order was then added to sex and species as fixed effects in an alternative model to the null, with sex and species only. The evidence for an effect of the added predictor was assessed by anova.lme command as described above. If the null hypothesis of no effect of the predictor was rejected with *P* < 0.05 then the predictor was retained, and a new model was estimated with the second predictor added to the fixed effects as last-named in the sequence. If the null hypothesis for the first predictor was accepted, then it was dropped from the fixed effects, and a new model was fitted with the second predictor added to sex and species as fixed effects. This procedure was iterated until all the predictors had been considered.

The objective of this model fitting was to identify measures of intake that may be predictive of elemental status. The inference in the procedure above from p-values is properly done to test single hypotheses, and the procedure here is multiple hypothesis testing in which our question was ‘are any of the measures of intake predictive?’. It is well known that multiple hypothesis testing in which each hypothesis is evaluated on the basis of its own *p-valu*e is likely to be anticonservative, in the sense that the final set of selected predictors is likely to contain some, which result from the rejection of null hypotheses, which should have been accepted. Here we used the control of marginal discovery rate to control the error, based on the approach of Benjamini and Hochberg (1995)^38^. The false discovery rate is the probability that a rejected null hypothesis in some family of tests should have been accepted. We controlled marginal false discovery rate (mFDR) at <0.05, following the method of alpha-investment proposed by Foster & Stine (2008)^39^.

Under Foster and Stine’s (2008)^39^ procedure, the p-values for a set of tests conducted in some order are compared against a set of threshold values. The threshold value for the *i*^th^ test is not fixed in advance but depends on a quantity called the alpha-wealth, which is depleted (by acceptance of null hypotheses at positions 1 to (*i-*1) in the sequence) or augmented by rejection of these hypotheses. Foster and Stine (2008)^39^ present rules for the development of the alpha wealth, which ensure that the false discovery rate is controlled below the specified value. These mean that judicious ordering of the hypotheses, so that the least plausible nulls are tested early, (i.e. the predictors most likely to be informative) will increase the power of the procedure to detect real effects (although this is not valid if the ordering is based on prior examination of the data). More detail of the theory of this procedure is presented by Foster and Stine (2008)^39^ and Lark (2017)^40^ gives an example of its application. It has been used in various studies to improve the efficiency with which large data bases are interrogated to identify effects of interest while controlling false discovery rate^41^.

The p-values from the successive tests of null hypotheses for each element and sample matrix were evaluated against threshold values determined by the method of Foster and Stine (2008)^39^, specifying that the false discovery rate be kept below 0.05.

The process above results in a predictive model for a mineral in a particular sample matrix, with selected predictors related to possible sources of intake, or no predictors are selected. In the former case we compute a measure of the extent to which the predictive model succeeds in accounting for variation in the observed concentrations in the sample matrix of interest. This is called the approximate adjusted R^2^ and it is computed for each random effect in the model: the between-zoo, between-elephant within zoo and between observations within elephant random effects (the latter including measurement error). If the estimated between-zoo variance component for the null model, with sex and species the only fixed effects, is *s*^2^_Z,0_, and the corresponding variance component for the model with additional selected predictors is *s*^2^_Z,1_, then the approximate adjusted R^2^ at between-zoo level may be computed as

1 $${R}_{Z}^{2}=1-{s}_{Z,1}^{2}/{s}_{Z,0}^{2}.$$

We may think of this quantity as the approximate proportion of variance (at the between-zoo level) accounted for by adding the extra predictors to sex and species. It should be noted that, unlike the R^2^ values customarily computed with statistical software, this one is based on likelihood-based estimates of variance components rather than those obtained by partition of a sum of squared residuals. It is therefore possible that an estimated variance component could be negative, due to estimation error^42^, and so the value computed in Eq. [1] might not be bounded by [0,1]. Negative values simply imply that the effect of adding the predictor, at this level, is smaller than the estimation error, and a value of 1 implies that the variance component in the model with predictors is very small and has been estimated as zero as (or less than zero). A value of *R*^2^_Z_ < 0 should therefore be interpreted as evidence that the predictor has negligible effect in terms of accounting for variation in the observations at the between-zoo level, and a value of 1 that it accounts for most of the variation at this level.

## Results

### Overall results

Tables 2 and 3 summarise results of all intakes sampled (keeper-fed diet, grass, soil and water) at the five study zoos. All individual data points are shown in Supplementary Information Tables 2 and 4 to 8.

**Table 2:** Median summary data for inputs (this includes keeper-fed diet and pasture grass) to all elephants at each of the five UK zoos (A, D, E = *Loxodonta africana*, B, C = *Elephas maximus*) at the four collection time points.

Zoo		units	Keeper-fed diet - as fed					Grass - estimated input, as fed					
			A	D	E	B	C	units	A	D	E	B	C
			*L. africana*			*E. maximus*			*L. africana*			*E. maximus*	
		Number elephants (m/f)	4 (0/4)	4 (1/3)	2 (2/0)	4 (0/4)	7 (1/6)		4 (0/4)	4 (1/3)	2 (2/0)	4 (0/4)	7 (1/6)
Ca	median	g	312	529	340	147	327	g	33	72	12	261	37
	IQR		156	190	59	66	40		34	56	7	127	38
	n		16	16	8	14	28		16	16	8	6	26
Mg	median	g	73	157	76	53	118	g	17	17	3	141	10
	IQR		15	28	13	19	7		14	14	5	16	12
	n		16	16	8	14	28		16	16	8	6	26
Na	median	g	49	107	65	41	207	g	4	4	1	6	4
	IQR		24	64	49	27	68		4	3	1	1	3
	n		16	16	8	14	28		16	16	8	6	26
P	median	g	121	215	143	86	128	g	19	35	5	68	17
	IQR		8	111	9	27	68		21	20	7	97	20
	n		16	16	8	14	28		16	16	8	6	26
K	median	g	736	1488	929	579	460	g	126	203	40	524	129
	IQR		36	622	163	54	48		125	98	41	755	171
	n		16	16	8	14	28		16	16	8	6	26
Cu	median	g	0.9	1.3	0.8	0.6	0.8	mg	373	636	30	1063	255
	IQR		0.8	0.6	1.1	0.6	1.0		825	620	61	655	514
	n		16	16	8	14	28		16	16	8	6	26
Fe	median	g	8	18	9	6	11	g	35	13	6	215	8
	IQR		3	15	2	3	12		56	18	13	304	16
	n		16	16	8	14	28		16	16	8	6	26
Mn	median	g	7.6	8.0	3.8	1.6	12	g	17	2	0	6	1
	IQR		5.1	6.8	2.4	0.7	6.3		4	3	2	4	1
	n		16	16	8	14	28		16	16	12	6	26
Se	median	mg	2.4	13.0	2.9	1.5	3.2	mg	1.2	0.9	0.2	3.0	0.5
	IQR		0.7	7.1	0.3	1.1	0.8		1.4	0.06	0.2	1.7	1.0
	n		16	16	8	14	28		16	16	8	6	26
Zn	median	g	3.2	5.0	3.1	1.0	2.2	mg	386	482	85	1529	229
	IQR		1.8	1.6	0.6	0.4	0.3		422	501	193	476	257
	n		16	16	8	14	28		16	16	8	6	26
As	median	mg	4	13	8	2	4	mg	18	6	4	78	3
	IQR		0.9	7.6	3.5	1.0	4.9		30	9	9	96	6
	n		16	16	8	14	28		16	16	8	6	26
Cd	median	mg	39	11	10	3	8	mg	5.0	0.6	0.6	5.0	0.4
	IQR		32	10	40	3	4		7.2	0.9	1.0	2.0	0.5
	n		16	16	8	14	28		16	16	8	6	26
Pb	median	mg	24	56	29	9	22	mg	121	20	13	323	12
	IQR		27	30	33	4	35		181	30	29	399	22
	n		16	16	8	14	28		16	16	8	6	26
U	median	mg	4	8	6	3	8	mg	1	0	0	8	0
	IQR		1	5	1	10	1		2.1	0.6	0.5	11.4	0.4
	n		16	16	8	14	28		16	16	8	6	26
V	median	mg	13	48	20	11	24	mg	68	24	10	397	18
	IQR		5	45	3	12	23		112	41	24	552	36
	n		16	16	8	14	28		16	16	8	6	26

CRM data for all diet items can be found in Supplementary Information Table 2. Keeper-fed diets n = number of diets (consisting of multiple feed items) at zoo, grass n= number grass samples collected.

**Table 3:** Summary data for inputs (water and soil) to all elephants at each of the five UK zoos (A, D, E = *Loxodonta africana*, B, C = *Elephas maximus*), samples were taken at the first visit. CRM data can be found in Supplementary Information Table 3.

	Water - based on estimated input of 200 litres per elephant per day						Soil - Dry Matter					
Zoo		A	D	E	B	C	units	A	D	E	B	C
Species	units	*L. africana*			*E. maximus*			*L. africana*			*E. maximus*	
Element	Number elephants (m/f)	4 (0/4)	4 (1/3)	2 (2/0)	4 (0/4)	7 (1/6)		4 (0/4)	4 (1/3)	2 (2/0)	4 (0/4)	7 (1/6)
Ca	g	4	22	18	11	22	mg/kg	3369	1742	8045	10044	3354
Mg	g	0.5	3.4	2.3	2.1	0.8		4194	1352	5272	14985	2398
Na	g	2.8	10.9	3.5	3.9	3.5		3206	2054	2171	3437	3992
P	mg	322	356	366	4200	4		1086	298	790	493	817
K	g	0.2	1.7	0.5	2.6	0.7		13323	9397	27705	24989	10771
Cu	mg	0.1	12	3	1.6	0.6		76	11	19	16	19
Fe	mg	3	0	15	20	11		23600	15565	33053	26721	27092
Mn	mg	0.0	0.1	2.2	13.0	0.8		595	152	1883	462	955
Se	mg	0.0	0.6	0.1	0.1	0.1		0.63	0.17	0.54	0.25	0.45
Zn	mg	1	2	144	6	109		101	36	191	91	81
As	mg	22	44	342	56	36		11	7	28	9	123
Cd	mg	2	2	32	6	2		2.0	0.1	1.4	0.2	0.4
Pb	mg	0	14	252	12	32		74	12	87	39	49
U	mg	2	138	81	88	48		1.7	0.9	2.5	1.6	1.8
V	mg	20	20	140	40	40		61	36	70	62	74

### Elemental data for intakes (diet, grass, water and soil)

Based on observations of the elephants, and keeper feedback, on all four visits to each of the five zoos, it was clear that the elephants consumed all the food presented to them in their keeper-fed diets, over a 24-hour period. At all five zoos, hay made up the largest contribution to the diet by weight, on average 52 kg per elephant per day (+/− 15 kg) out of an average keeper-fed diet of 81 kg per elephant per day (over 60% of intake), other than possible grazing for which accurate quantification was not possible. Seasonally, there was little variation in mineral provision from the hay and amounts fed were consistent throughout the year in all but one zoo (Zoo C), as shown in Supplementary Information Tables 2–8.

Commercial pellets were the main source of dietary minerals. Across the five zoos, 10 different commercial pellets were fed with four out of the five zoos feeding a pellet that was manufactured specifically for the species. One pellet had a Zn supplement milled into it (fed at Zoo C) and was fed to specific individuals that were suspected to be Zn deficient from previous in house assessment. All but one zoo fed wheat bran to their elephants in minimal quantities as a medicant carrier. Five additional nutraceutical or vitamin/mineral powdered supplements were added to the keeper- fed diets across three zoos (Zoos A, C and D): Bladder-rite (Gold Label, UK), Newmarket Joint Supplement (Newmarket, UK), multivitamin supplement (Farm and Stables Supplies, UK), multivitamin with additional vitamin E and Se supplement (Farm and Stables Supplies, UK), and calcium carbonate (CaCO_3_) (Farm and Stables Supplies, UK). Two zoos provided no additional supplements (Zoos B and E).

Grass from pasture grazing was not available to all elephants throughout the year, as access depended on the weather conditions; however, all zoos offered grazing to all animals for at least part of the year (generally in spring, summer or autumn). Mineral content in grass from pasture grazing, and from browse consumption, varied considerably across seasons and between zoos. Generally, less browse was presented to elephants during winter months due to the challenges of sourcing palatable material in the UK climate. Fruit and vegetables comprised a very small proportion of all elephant diets (by weight) despite the wide variety of items fed (18 varieties of vegetables and 9 varieties of fruit over the five zoos), thus contributed minimally to the mineral provision in the diet due to the very small quantities fed per day and their high water content.

**Figure 1:**
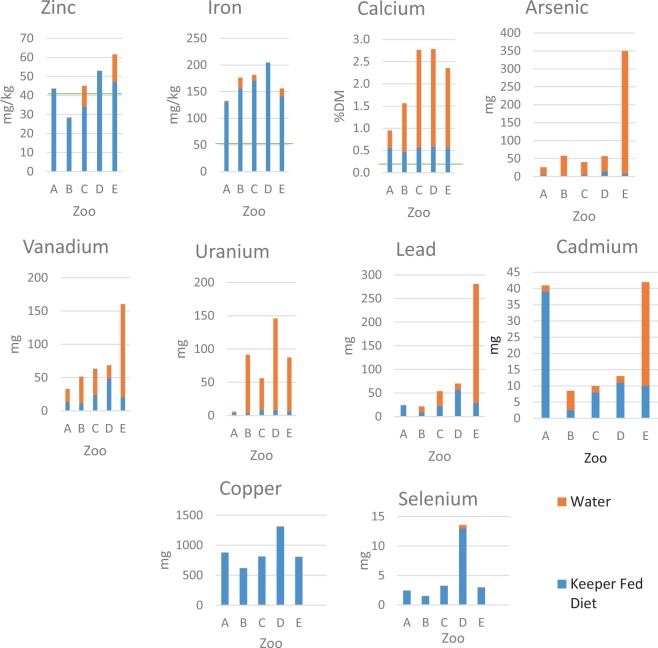
Median dietary element intake per zoo from keeper-fed diets and water (estimated at 200 litres per animal per day), in line with dietary recommendations^34^, as shown by the green line.

For certain minerals, provision from water contributed substantially to the overall mineral intake for the elephants, with considerable variation between zoos noted, as shown in Fig. 1. Specific elements of interest provided through drinking water included Zn where provision in zoos C and E were noteworthy, Ca in all the study zoos, and As and U in which provision from water contributed to intake more than keeper-fed diets. Additionally, at Zoo E, levels of Cd, Pb and V in the water contributed substantially to overall intake of these elements at this zoo. All water samples fell within the safe water limits issued for humans by the World Health Organization (WHO 2018)^43^.

It was not possible to quantify elemental provision from soil, although anecdotally, keepers reported that elephants were seen to consume soil in very small quantities on occasion. Table 3 indicates that there is considerable variation in elemental provision from soil at the various zoos. This is likely to be due to geographical location of each zoo and geological makeup of the associated soil^44^.

### Dietary provision compared to published recommendations

Calcium, Fe and Zn all have published recommended dietary intakes for elephants^4^. Figure 1 shows a comparison of mineral levels in keeper-fed diets from each zoo to captive recommendations produced by Ullrey *et al*.^34^. In all zoos, Ca and Fe concentrations in provisioned diets were well in excess compared with intake recommendations, whereas Zn was below the recommended levels in keeper-fed diets in two of the five zoos. However, when Zn provision from water was also considered (based on assumed consumption of 200 litres per elephant per day^11,31,32^), the Zn level in Zoo C rose to within the recommended levels and only intake in Zoo B remained below the recommended level of 40 mg/kg (DM basis).

### Elemental reflection in elephant sample matrices

Tables 4 and 5 and Supplementary Information Table 10, summarise the results of all outputs from elephants at the five study zoos (toenail, tail hair, faeces, urine and plasma), divided into fluids and solids. Urine elemental analysis was corrected for hydration status using creatinine normalization^30^. Elephant tail hair is estimated to grow at approximately 2–3.5 cm per month, with two studies of 50 and 32 hairs, respectively, documenting this growth rate and reported that tail hair of males grew up to 1.5 cm more slowly per month than those of females^20,45^.

**Table 4:** Median summary elemental data for sample matrices (fluid outputs) from elephants at all UK zoos; this includes plasma and urine (corrected for hydration status using a ratio with creatinine), from all sampled elephants (21 individuals) at each of the five UK zoos (A, D, E = *Loxodonta africana*, B, C = *Elephas maximus*), at the four collection time points.

Zoo	Median plasma						Median Urine: creatinine		
	units	A	D	E	B	C	Ratio	D	C
Species		*L. africana*			*E. maximus*			*L. africana*	*E. maximus*
Number elephants (m/f)		4 (0/4)	4 (1/3)	2 (2/0)	4 (0/4)	7 (1/6)		3 (1/2)	6 (0/6)
Number samples		9	16	4	5	28		11	21
Ca	mg/l	107	105	104	86	108	Ca:Cre	2.3	5
Mg	mg/l	28	25	26	22	28	Mg:Cre	0.6	1.7
Na	mg/l	2955	3038	3109	2603	3218	Na:Cre	0.1	2.1
P	mg/l	91	81	95	67	78	P:Cre	0.01	<0.001
K	mg/l	284	205	192	178	182	K:Cre	5.6	19
Cu	ug/l	919	988	1001	736	1096	Cu:Cre	<0.001	<0.001
Fe	ug/l	1.6	3.2	0.9	1.5	1.1	Fe:Cre	<0.001	<0.001
Mn	ug/l	2.1	1.6	3.8	2.3	1.5	Mn:Cre	<0.001	<0.001
Se	ug/l	125	162	118	106	187	Se:Cre	<0.001	<0.001
Zn	ug/l	1399	1056	1129	614	737	Zn:Cre	<0.001	<0.001
As	ug/l	0.5	0.8	1.0	0.3	0.5	As:Cre	<0.001	<0.001
Cd	ug/l	0.2	0.06	0.08	0.1	0.03	Cd:Cre	<0.001	<0.001
Pb	ug/l	0.1	0.48	0.81	0.2	0.18	Pb:Cre	<0.001	<0.001
U	ug/l	0.02	0	0.00	0.02	0.00	U:Cre	<0.001	<0.001
V	ug/l	0.23	0.22	0.22	0.22	0.28	V:Cre	<0.001	<0.001

All data reported on a wet basis, see Supplementary Information Table 10 for further data points.

**Table 5:** Median summary elemental data for sample matrices (solid outputs); this included faeces, toenails and tail hair from all sampled elephants (21 individuals) at each of the five UK zoos (A, D, E = *Loxodonta africana*, B, C = *Elephas maximus*), at the four collection time points.

Zoo		units	Faeces dry matter					Toenail					Tail hair				
			A	D	E	B	C	A	D	E	B	C	A	D	E	B	C
Species			*L. africana*			*E. maximus*		*L. africana*			*E. maximus*		*L. africana*			*E. maximus*	
Number animals (m/f)			4 (0/4)	4 (1/3)	2 (2/0)	4 (0/4)	7 (1/6)	4 (0/4)	4 (1/3)	2 (2/0)	4 (0/4)	7 (1/6)	4 (0/4)	4 (1/3)	2 (2/0)	4 (0/4)	7 (1/6)
Ca	median	mg/kg	4229	3887	7758	2764	3615	2355	3807	3716	4496	3296	2958	3155	1658	2046	3118
	n		16	16	8	10	27	16	16	8	15	28	20	13	6	5	29
Mg	median	mg/kg	1013	1330	1442	1230	1620	224	587	304	456	224	450	422	198	230	175
	n		16	16	8	10	27	16	16	8	15	28	20	13	6	5	29
Na	median	mg/kg	1522	2766	2446	2384	4415	37	108	180	88	57	351	132	390	218	99
	n		16	16	8	10	27	16	16	8	15	28	20	13	6	5	29
P	median	mg/kg	2709	2946	4337	2895	3680	57	106	93	57	76	65	159	63	102	79
	n		16	16	8	10	27	16	16	8	15	28	20	13	6	5	29
K	median	mg/kg	13408	11625	14654	11965	10738	252	874	350	825	225	1643	301	202	1073	126
	n		16	16	8	10	27	8	16	8	11	28	20	13	6	5	29
Cu	median	mg/kg	18	30	29	28	30	1.8	2.4	2.2	1.1	2	17	15	14	10	11
	n		16	16	8	10	27	16	16	8	15	28	20	13	6	5	29
Fe	median	mg/kg	735	1428	1651	645	562	247	730	463	356	351	58	0	17	314	93
	n		16	16	8	10	27	16	16	8	15	28	15	13	6	5	29
Mn	median	mg/kg	197	143	174	80	295	14	18	25	11	21	29	13	36	13	37
	n		16	16	8	10	27	16	16	8	15	28	20	13	6	5	29
Se	median	mg/kg	0.05	0.16	0.1	0.03	0.1	0.3	0.6	0.2	0.4	0.4	1.0	1.5	0.8	1.0	0.2
	n		16	16	8	10	27	16	16	8	15	28	20	13	6	5	29
Zn	median	mg/kg	76	68	89	53	61	51	55	39	39	30	125	94	108	94	101
	n		16	16	8	10	27	16	16	8	15	28	20	13	6	5	29
As	median	mg/kg	0.3	0.6	1.2	0.3	0.3	0.2	0.8	0.8	0.3	0.4	0.2	0.2	0.2	0.3	0.1
	n		16	16	8	10	27	16	16	8	15	28	20	13	6	5	29
Cd	median	mg/kg	1.90	0.12	0.35	0.20	0.15	0.05	0.02	0.07	0.05	0.02	0.12	0.02	0.05	0.09	0.05
	n		16	16	8	10	27	16	16	8	15	28	20	13	6	5	29
Pb	median	mg/kg	2.7	1.85	3.78	1.2	1.2	0.48	0.66	1.87	1.03	0.49	0.45	0.48	0.45	1.02	0.25
	n		16	16	8	10	27	16	16	8	15	28	20	13	6	5	29
U	median	mg/kg	0.20	0.19	0.38	0.10	0.34	0.01	0.03	0.02	0.03	0.02	0.002	0.012	0.003	0.010	0.004
	n		16	16	8	10	27	16	16	8	15	28	20	13	6	5	29
V	median	mg/kg	1.65	3.85	3.37	1.2	1.59	0.37	1.42	0.84	0.41	0.87	0.10	0.3	0.00	0.50	0.10
	n		16	16	8	10	27	16	16	8	15	28	20	13	6	5	29

All data reported on a dry matter basis. Further data points are reported in Supplementary Information Table 10.

These data reported for zoo elephants are therefore assumed to represent the median elemental analysis for approximately the most recent 6 months of growth: 3 × 3 cm sections for males and 5 × 3 cm sections for females, given that growth rate of males is documented to be slower than females.

### Identifying best sample matrices for each element

Table 6 summarises results from the linear mixed model, and the significance of correlation with combined inputs, including the keeper-fed diet, estimated grass consumption and estimated water consumption. Toenail was found to best reflect Na and K intakes. Iron intake was best reflected in both tail hair and toenail samples. Phosphorus and Ca levels in faeces correlated with combined inputs and Se intake correlated with toenail and faecal levels. Finally, Cd and U intake were reflected in plasma samples. Elemental levels in urine were found to have no significant correlation with any combined inputs.

**Table 6:** **Linear** mixed model p-values of combined input values compared to sample matrices.

Element		Tail hair	Toenail	Plasma	Faeces
Ca	p-value	0.513	0.159	0.992	**0.019**
	R^2^E				0.608
	R^2^O				0.092
Mg	p-value	0.450	0.599	0.357	0.924
Na	p-value	0.076	**0.039**	0.309	0.136
	R^2^E		<0.000		
	R^2^O		0.061		
P	p-value	0.318	0.165	0.812	0.012
	R^2^E				<0.000
	R^2^O				0.142
K	p-value	0.791	**0.031**	0.869	0.411
	R^2^E		1.000		
	R^2^O		<0.000		
Cu	p-value	0.467	0.445	0.475	0.671
Fe	p-value	**0.029**	**0.012**	0.390	0.582
	R^2^E	0.406	<0.000		
	R^2^O	0.256	0.149		
Mn	p-value	0.630	0.707	0.650	0.498
Se	p-value		**0.031**	0.887	**0.000**
	R^2^E		0.186		0.521
	R^2^O		<0.000		0.077
Zn	p-value	0.824	0.502	0.068	0.691
As	p-value	0.175	0.237	0.644	0.511
Cd	p-value	0.088	0.975	**<0.0001**	0.907
	R^2^E			<0.000	
	R^2^O			0.287	
Pb	p-value	0.224	0.397	0.485	0.394
U	p-value	0.356	0.124	**0.004**	0.552
	R^2^E			<0.000	
	R^2^O			0.150	
V	p-value	0.090	0.412	0.288	0.481

Combined inputs include keeper-fed diet, estimated elemental provision from water and grass consumption based on estimated intakes. Urine is not included as there was inadequate balanced replication to estimate model. R^2^ E = between-elephant within zoo and R^2^ O = between observations within elephant random effects (the latter including measurement error).

Table 7 shows summary results from within the linear mixed model to identify the best sample matrices for reflecting bio-indicators of intake and therefore a good proxy of elemental status. The R^2^ value (in Tables 6 and 7) considers other factors which may influence the significant predictor; R^2^ E = between elephant within zoo and R^2^ O = between observations within elephant random effects (the latter including measurement of error). This value increases confidence towards the predictor. Supplementary Information Table 11 details all p-values resulting from this model. Inputs were considered sequentially, with diet as the first predictor followed by grass, water and finally soil. Toenail was found to reflect the greatest number of elements, followed by faeces and blood. Tail hair and urine reflected the least number of elements. Diet was the most significant predictor for the greatest number of elements (8) followed by grass, soil and water. Due to the reduced sample size for urine, there is inadequate balanced replication to estimate the full model, so sex and species effects were removed as random effects. No significant predictors were found for Cd, Na, Pb and V.

**Table 7:** Linear mixed model results to identify bio-indicators of elemental status depending on multi-layered model using diet, grass, water and soil as predictors.

Element		Tail hair	Toenail	Plasma	Urine	Faeces
Ca	predictor	no significant predictors				diet
	R^2^E					0.919
	R^2^O					0.085
Mg	predictor	no significant predictors	diet	no significant predictors		
	R^2^E		<0.000			
	R^2^O		0.212			
	predictor		soil			
	R^2^E		0.175			
	R^2^O		0.227			
P	predictor	no significant predictors	soil	no significant predictors		
	R^2^E		0.320			
	R^2^O		<0.000			
K	predictor	no significant predictors	diet	no significant predictors		
	R^2^E		1.000			
	R^2^O		0.146			
	predictor		grass			
	R^2^E		<0.000			
	R^2^O		0.586			
Cu	predictor	no significant predictors				diet
	R^2^E					<0.000
	R^2^O					0.063
Fe	predictor	no significant predictors		soil	no significant predictors	
	R^2^E			0.588		
	R^2^O			0.030		
Mn	predictor	no significant predictors		soil	no significant predictors	
	R^2^E			<0.000		
	R^2^O			<0.000		
Se	predictor	no significant predictors	diet	no significant predictors		diet
	R^2^E		0.140			0.663
	R^2^O		<0.000			0.103
Zn	predictor	no significant predictors	no significant predictors	diet	no significant predictors	no significant predictors
	R^2^E			<0.000		
	R^2^O			0.056		
As	predictor	grass	diet	no significant predictors		diet
	R^2^E	0.488	<0.000			<0.000
	R^2^O	0.093	0.159			0.115
U	predictor	no significant predictors		diet	no significant predictors	
	R^2^E			0.315		
	R^2^O			0.120		
	predictor			grass		
	R^2^E			<0.000		
	R^2^O			0.490		

R^2^ E = between-elephant within zoo and R^2^ O = between observations within elephant random effects (the latter including measurement error).

## Discussion

The aim of this study was to identify appropriate sample matrices to use as bio-indicators of elemental status in elephants. The variability in dietary provision by zoos presented a challenge, and the complexity of estimating the ‘inputs’, i.e. keeper-fed diet including browse, grass provision from grazing, and intake of water and soil, demonstrates the need to be able to simply obtain non-invasive samples for assessing elemental status in the species. A strong bio-indicator must respond well to variation in intake, so we examined relationships between elemental composition of sampled sample matrices and known inputs as shown in Tables 6 and 7.

Tables 6 and 7 demonstrate that non-invasive sampling of tail hairs and toenails can potentially provide useful indications of As, Fe, K, Mg, Na, P, Se, and Zn status in elephants. Results suggest that the current clinical practice of elemental measurement using plasma is of limited value, as such measures are rarely responsive to dietary variation. There were no useful biomarkers of intake for Cd, Na, Pb and V when considered sequentially and for As, Cu, Mg, Mn, Pb, V and Zn when considered on a combined input basis. Additionally, faeces reflected As, Ca, Cu and Se intake on a sequential basis and Ca, Se and P on an estimated combined input basis. Manganese and U intakes were reflected in blood sequentially. Where there is no elephant-derived sample that can indicate intake of an element, zoos may find it useful to be aware of alternative measures (e.g. haemoglobin status for Fe) and consider the elemental composition of their water sources.

Toenails were found to best reflect elemental intake for the largest number of elements in elephants: As, Fe, K, Mg, Na, P and Se as shown in Tables 6 and 7. Unfortunately, it was not possible to estimate the growth rate of the toenails or the time during which the material analysed was laid down by the elephant. A number of factors including substrate, exercise, weather, foot care, nutritional and health status of the animal, as well as behaviour will affect toenail growth rates^46^. Tail hair proved to be a bio-indicator of total intake of key minerals, including As and Fe. Tail hair growth rate, as with other mammals, is also likely to be affected by multiple factors including overall nutritional plane, weather, health of the elephant and age of the elephant^47^. Therefore, estimated growth rate was used to calculate the analysed length^20,45^, which is an estimation of the previous 6 months of growth, although in practice this may not always be accurate due to variability in growth rate. Faecal samples were indicative of estimated elemental intake in the elephant for As, Ca, Cu, Se and P, as shown in Tables 6 and 7. Due to historic concern around insufficient vitamin E and other antioxidants in zoo elephant diets, Se is likely being fed in excess as an additive within the manufactured elephant pellets^48^. This could indicate why high concentrations were excreted and therefore detected within faecal samples.

Macro-mineral concentrations within blood plasma were not found to be reflective of intake. This is unsurprising, as mammals homeostatically control levels of these minerals within the blood and store excess as needed^12,14,15^. Urine was not found to indicate estimated intake of any element. It was only possible to obtain 32 samples from 9 animals at two zoos. Therefore, there was inadequate balanced replication to estimate the full model, thus sex and species effects were removed.

Generally, UK zoo elephants are not mineral deficient, and often for several minerals, keeper-fed diets contain excess provision. For specific elements such as Pb, inputs were very low (in keeper-fed diet, water, soil or grass) and thus, Pb was not present in sufficient quantities in any inputs, to be reflected in any elephant samples. A linear mixed model with the application of alpha wealth was appropriate to identify significant relationships between inputs and sample matrices (outputs) both on a combined and sequential method. The use of alpha wealth to reduce the false discovery rate within the linear mixed model provided greater confidence in the findings; nine previously significant relationships were eliminated through this correction. The application of R^2^ allows for consideration to be given to the weighting of factors other than the one being investigated that may have affected relationships, including between-elephant within zoo and between observations within elephant random effects (the latter including measurement error). Elemental combined input based on keeper-fed diet, estimated water consumption (200 litres/day) and estimated grass intake from grazing (based on %DM consumption), resulted in less significant relationships with sample matrices than when considered sequentially. This is likely because estimations were made of inputs, especially of grass intake, to form the combined input figure.

Keeper-fed diet was the most likely predictor of elemental values measured in any sample matrix and was the major source of minerals, as shown in Table 7. From this source, the pellet ration within the diet contributed most to elemental provision, even though the weight of pellet fed in the overall keeper-fed diet was comparatively low, on average 9% of the diet as fed (an average 7 ± 9 kg per elephant per day). However, only Zoo B and C fed less than the recommended 3 kg of pellet per day^49^. Variation in elemental provision from hay throughout the year was minimal, mineral or trace metal degradation would not be expected with storage. Hay and browse used within each zoo were produced within close proximity (within approx. 20 km) of each zoo. The variation in elemental analysis in hay and browse between zoos, is likely to reflect the differing geology of the five areas surrounding the zoos.

Water mineral concentrations varied widely both among zoos and seasonally, often depending on source / availability, e.g. borehole, rainwater harvesting or mains water supply. Elemental provision from water is rarely considered as significant when evaluating human or animal diets, except when investigating exposure to trace metals. However, Fig. 1 shows that water contributed substantially to the intake of specific minerals (As, Ca, Cd, Pb, U, V and Zn, Cd) and requires further study. At zoo C, Zn provision from keeper-fed diet was below the recommended levels. With the addition of Zn from the water provided (assuming average levels of water intake from the literature, 200 litres /day), apparent levels of Zn became sufficient. Beal (2017)^50^ demonstrated the significance of Ca provision in human drinking water, where Ca from water contributed up to 11% of national Ca supplied, based on consumption of 1.7 litres per adult per day. Within this study, Ca in water was found to contribute up to 8% of Ca supply per day, based on an estimated consumption of 200 litres of water per elephant per day.

Zoos were selected to provide a geographical and geological spread with variance within the soil make up, therefore the variation seen in the elemental analysis of soils sampled was not unexpected. The linear mixed model determined that soil minerals were a significant predictor of Mg and P levels in toenail and Fe and Mn in plasma. For these elements, provision from soil may be important to consider when looking at elemental reflection in these sample matrices.

There are limited published recommendations for elephant dietary mineral provision. Generally the domestic horse is considered to be an acceptable physiological model for elephants^4,51^. Additionally, the BIAZA (British and Irish Association of Zoos and Aquaria) Elephant Management Guidelines (2019)^4^ provides recommendations on dietary management, which must be followed by BIAZA member zoos as part of the BIAZA Elephant Management Policy. All zoos within this study fed browse daily throughout the year and provided some grazing access to all animals, in line with these BIAZA recommendations.

Mineral provision from the keeper-fed diet within this study was found to be similar to previous work. Partington (2012)^52^ found all UK elephant diets to be excessive in Ca, as was the case in this study, and also detected some possible Zn deficiencies. The study conducted by Partington used intake data from all UK elephant holding zoos at the time of writing but used published elemental data for each food item and did not include elemental provision from browse as was included in the current study. The lowest Zn dietary intake identified by Partington (2012)^52^ was 22 mg/kg DM, whereas in this study, the lowest Zn dietary intake was 28 mg/kg DM (Zoo B) as shown in Fig. 1, but still below the recommended 40 mg/kg DM.

Published reference ranges for elephant plasma, urine and faecal mineral levels are very limited. The largest database of this information resides with Species 360, USA^27^. All member zoos internationally are encouraged to submit data as available. Sample sizes of these datasets are often small and animals may be health-compromised at the time of sampling. Results for elemental levels in plasma, urine and faeces in this study (Tables 4 and 5 and Supplementary Information Table 10) were within reported reference ranges, Species 360, 2017^27^. Published data on elemental analysis in elephant tail hair or toenails is absent from the literature.

Feed costs are the second largest day-to-day running costs of a captive elephant herd^4,22^. It is therefore essential that zoos are feeding appropriate items of acceptable quality to their animals. The BIAZA Elephant Management Guidelines recommends the use of fruits and vegetables in very limited quantities, less than 1 kg per elephant per day^53^. All zoos in the current study were feeding greatly in excess of this recommendation as shown in Supplementary Information Tables 4–8. Given the high incidence of obesity in UK zoo elephants, with estimations of up to 75% of the population being recorded as ‘overweight’^54^, a seasonal reduction in hay is recommended to offset increased grass from pasture and browse availability during summer months, this is currently only practiced in Zoo C.

Finally, the elephants in this study are under human care, and could arguably be variably stressed or compromised^55^, which may alter mineral metabolism. For example, plasma Zn levels can be artificially increased when an animal is stressed or suffering from an inflammatory condition^13^. Caution must be used when comparing these values to wild elephants, or as target values for elephants. Likewise, as seen within this current study, UK zoo elephants are unlikely to be experiencing nutritional compromise or substantial mineral deficiency. Diets fed in these five UK zoos, in general, were appropriate to meet species’ documented mineral needs.

## Conclusion

The results from the current study indicate that no single sample matrix from elephants are sufficient to reflect elemental intake within the animal, and thus be a good proxy for elemental status, a variety of sample matrices are needed. Of the five sample matrices investigated in this study, toenail reflected inputs for the largest number of elements assessed, and is likely to be the best reflection of status for these elements. Faeces and tail hair were also found to significantly correlate to inputs into the elephant. Plasma was of limited value with a small number of elements being responsive to dietary variation. Urine did not correlate with any inputs for any element and thus was not a useful bio-indicator. Predicting how elemental status is reflected in various sample matrices presents a challenge, as the sample matrix concentrations may not be indicative unless levels are below an excess threshold. Sample availability may also influence sample matrix choice when investigating mineral status. Finally, mineral provision from water should never be overlooked when assessing zoo animal diets, especially for species that consume such large volumes as elephants.

Future work should investigate how the methods described in this study could be applied to free-living populations of elephants, especially those within smaller fenced reserves, to identify individuals with mineral deficiencies, or elephants exposed to uncharacteristically high levels of trace metal intake. Opportunities exist to address the United Nations Sustainable Development Goals (SDGs) 3 (Good Health and Well-Being), 15 (Life on Land) and 17 (Partnerships for the Goals) from this work. Advancing zoo animal health and welfare will increase opportunities to (re) connect people with nature and promote well-being through the visiting of zoos (SDG 3), with further potential for education and creation of livelihoods (SDG 4, 8). Secondly, application of this work provides the opportunity to protect ecosystems through benefitting wildlife management (SDG 15). Finally, global partnerships can be developed between North and South with the opportunity for studies on captive animals in a controlled environment to inform research and welfare of wild counterparts (SDG 17).

## Supplementary information

Supplementary information.
